# Human frozen-thawed blastocyst morphokinetics observed using time-lapse cinematography reflects the number of trophectoderm cells

**DOI:** 10.1371/journal.pone.0210992

**Published:** 2019-01-16

**Authors:** Takuya Iwasawa, Kazumasa Takahashi, Mayumi Goto, Mibuki Anzai, Hiromitsu Shirasawa, Wataru Sato, Yukiyo Kumazawa, Yukihiro Terada

**Affiliations:** Department of Obstetrics and Gynecology, Akita University Graduate School of Medicine, Akita University, Akita, Japan; Peking University Third Hospital, CHINA

## Abstract

Recent studies reported morphokinetic indices for optimal selection of embryos in assisted reproductive technology (ART). The morphokinetics in blastocyst stage include the collapse and re-expansion rates after thawing. However, evaluation methods using these morphokinetics have not been established, mainly because the underlying molecular mechanisms remain unclarified. In this study, we focused on the relationship between these morphokinetic observation of the blastocyst behaviour and the number of cells constituting the blastocyst. We evaluated 38 surplus human frozen-thawed blastocysts using time-lapse cinematography and recorded their expansion, contraction, and hatching. A total of 28 blastocysts expanded in culture (cross-sectional area ≥ 5,000 π μm^2^). In comparison to the ones that did not, the expanded group presented significantly more number of inner cell mass (ICM) and trophectoderm (TE) cells, which eventually develop into the fetus and placenta, respectively (ICM: Expanded 10.2 ± 6.3 vs. Non-Expanded 6.0 ± 12.3, *p* < 0.05; TE: Expanded 165.7 ± 74.8 vs. Non-Expanded 57.0 ± 29.4, *p* < 0.05). Moreover, a positive correlation was found between the expansion rate (up to 4 h) and the number of TE cells (*r* = 0.558, *p* = 0.0021). Additionally, blastocysts that hatched had a significantly higher number of TE cells than those that did not (hatching 225.2 ± 61.2 vs. no hatching 121.1 ± 48.6, *p* < 0.0001). The number of TE cells per unit of cross-sectional area correlated negatively with the contraction time (*r* = –0.601, *p* = 0.0007). No correlation between the number of ICM cells and these morphokinetics was detected. In conclusion, our study demonstrates that different morphokinetics of frozen-thawed blastocysts reflect the number of TE cells. The differentiation of blastocysts containing sufficient TE cells would be beneficial for implantation and prognosis of a subsequent pregnancy. Thus, evaluation of these morphokinetics can be an effective method to screen good embryos for ART.

## Introduction

In 1978, the first child in the world was born with the aid of in vitro fertilization/embryo transfer (IVF/ET). In IVF, fertilized embryos undergo cell division in vitro. After compaction, the embryo forms a blastocoel, which becomes a blastocyst composed of the inner cell mass (ICM) and the trophectoderm (TE). The blastocyst further expands and hatches, breaking the zona pellucida. ET is a technique used to transfer the embryo resulting from the IVF process into the uterus for pregnancy.

In recent years, assisted reproductive technology (ART) has been widely practiced throughout the world and has greatly aided infertile patients. In ART, selection of good embryos (i.e., those with a high possibility of resulting in a successful pregnancy) is important for shortening the treatment period and reducing the physical and mental stress of the patient. The Gardner grading system, based on the degree of expansion of the blastocyst as well as ICM and TE qualities, is widely used to select embryos in ART [1]. Recently, morphological indices have attracted great interest, particularly the extent of blastocyst expansion and the TE grade [2,3]. However, this type of visual-based evaluation of the embryos can be highly subjective [4]. In addition, the dynamic development of blastocysts cannot be determined using static images. A previous report showed that measuring the dynamic index-of-expansion rate of the blastocyst after thawing is useful [5]. Importantly, the emergence of an in vitro culture system for time-lapse cinematography (TLC) has enabled further detailed kinetic analysis of the developmental process of early-stage embryos [6]. New embryo-evaluation methods with TLC utilize chronological and objective indices [4], and embryo morphokinetics may aid in selection of euploid embryos [7]. However, the morphokinetics observed using TLC has not been established as an effective outcome indicator in ART [8,9]. In the blastocyst stage, morphokinetics observable with TLC show variable re-expansion rate and collapse. Blastocysts showing collapse have been associated with decreased pregnancy rates in ART [10], although this is controversial [11]. The mechanisms by which these morphokinetics differ are not clearly understood, making it difficult to interpret the blastocyst behavior and predict outcome of ART.

We hypothesized that the difference in developmental morphokinetics is related to the number of cells constituting the ICM and TE. The purpose of this study was to test this hypothesis using surplus human embryos. We performed immunostaining against the blastocyst stage markers, Oct4 and Cdx2 to accurately count the numbers of ICM and TE cells, respectively [12], and analyzed their relationship with the different morphokinetic observation of the human blastocyst behaviour.

## Materials and methods

### Ethical approval

This original research study was approved by the Ethical Committee of Akita University (Permission number: 1090). The methods were carried out in accordance with the relevant regulations on research of human sperm/ovum/fertilized eggs set forth by the Japan Society of Obstetrics and Gynecology.

### Embryo source

We used 38 surplus embryos recovered from 18 infertile patients at the Akita University Hospital between 2006 and 2014, after obtaining written informed consent and performing linkable anonymization. Because it is impossible to obtain human embryos for research purposes, we decided to use embryos that were no longer in clinical use and were about to be discarded. The mean patient age (± standard deviation, SD) at the time of embryo freezing was 35 ± 4.5 years. Infertility was due to endometriosis (n = 6), tubal infertility (n = 2), male infertility (n = 2), or unknown causes (n = 8). The embryos were obtained after conventional IVF (n = 33) and intracytoplasmic sperm injection (n = 5).

The embryos were cultured in vitro after fertilization and frozen using the Cryotop Safety Kit (Kitazato, Japan) at 5 or 6 days post-fertilization (dpf) when the blastocysts reached grade 3 or higher (expansion), based on Gardner grading. The embryo was defined as a good embryo if the Gardner grade was 4BB or higher. We thawed 38 frozen embryos using the Cryotop Safety Kit (Kitazato, Japan), according to the manufacturer’s 2007 instructions. Briefly, the blastocyst was warmed by placing the Cryotop in thawing solution (1 mol/L sucrose) for < 60 s at 37 °C and then into the dilution solution (0.5 mol/L sucrose) for 3 min. The warmed blastocysts were washed 4–5 times in washing solution (Ham’s F-10 + 20% serum) [13].

### Embryo culture

Embryos were cultured in Sequential Blast^™^ medium without phenol red (ORIGIO, Denmark), which was covered with mineral oil to prevent concentration by evaporation. Embryos were cultured at 37 °C in a time-lapse incubator (Primo Vision; Vitrolife, Sweden) with 5% O_2_, 5% CO_2_, and 90% N_2_. The culture medium was not replaced during the experiments.

### Time-lapse imaging and analysis

**Fig 1:**
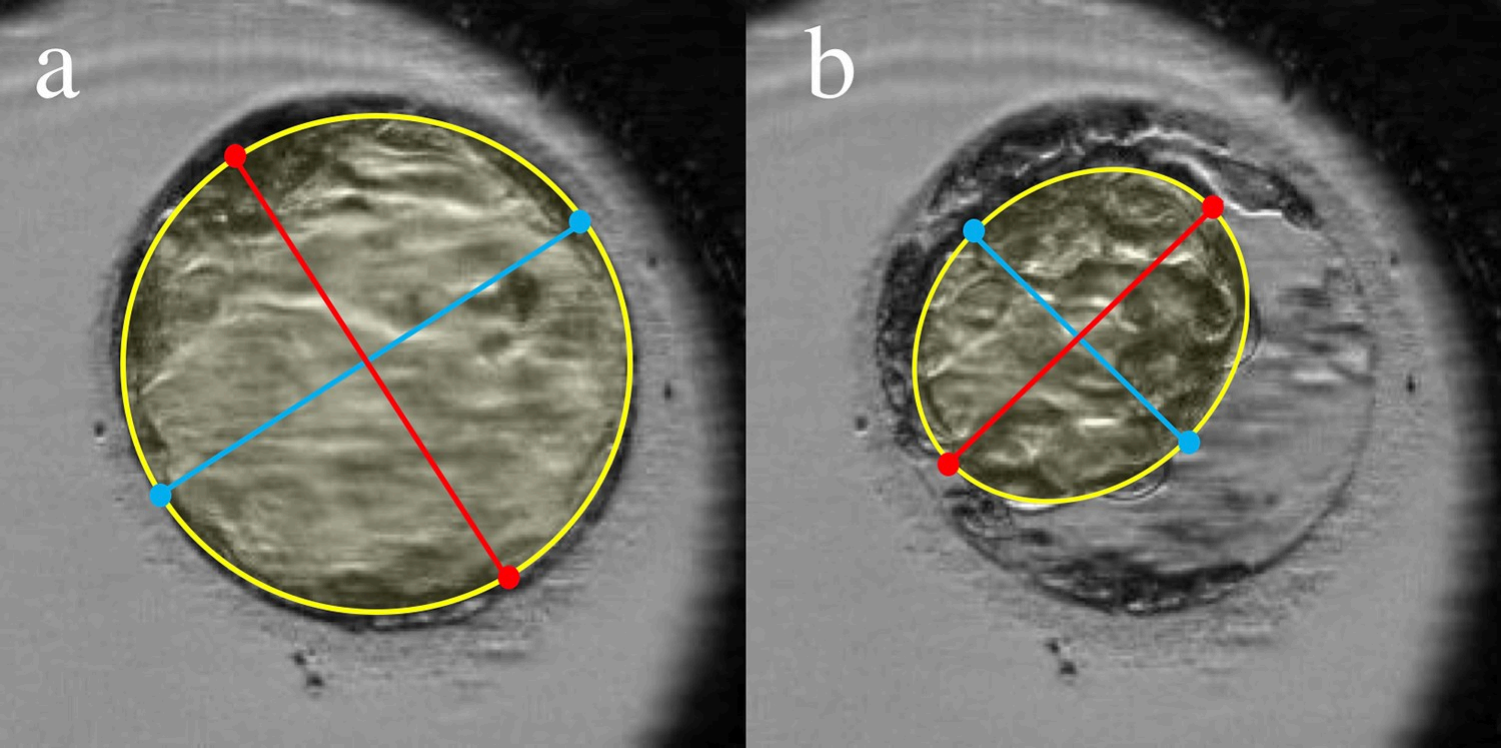
Measurement of the blastocyst cross-sectional area using TLC. The blastocyst cross-section is prominently elliptical (area outlined in yellow); the major axis (red) and minor axis (blue) were measured using ‘Primo Vision analyzer’ drawing tools. **(a)** Fully expanded blastocyst immediately before contraction. **(b)** Timepoint at which the cross-sectional area of the contracted blastocyst was the smallest.

Images for analyzing the morphokinetics of thawed embryos were acquired every 5 min for 22 h using TLC (Primo Vision). At the end of the initial expansion and every 4 h from the start of imaging, we recorded the cross-sectional area, the cross-sectional area before and after contraction, and the cross-sectional area at the start of hatching as well as the corresponding time. The cross-sectional area was calculated as the (long axis/2) × (short axis/2) × π, and the ‘Primo Vision analyzer’ drawing tools were used to calculate the cross-sectional areas for blastocyst cross-sections not containing a zona pellucida or that were prominently elliptical (Fig 1). We divided the blastocysts into two groups: the Expanded and Non-Expanded groups. The maximal cross-sectional area of the blastocysts in the Expanded group was 5,000 π μm^2^ or higher within 22 h of thawing. We determined the expansion rate of the blastocyst cross-sectional area up to 4 h from the start of imaging for blastocysts in the Expanded group and used this value as the initial expansion rate. For blastocysts that contracted or hatched within 4 h, the expansion rate up to that point was set as the initial expansion rate. We recorded the initial expansion rate, maximum expansion area, number of contractions, and hatching occurrence for each blastocyst.

### Definition and measurement/annotation of blastocyst contraction

**Fig 2:**
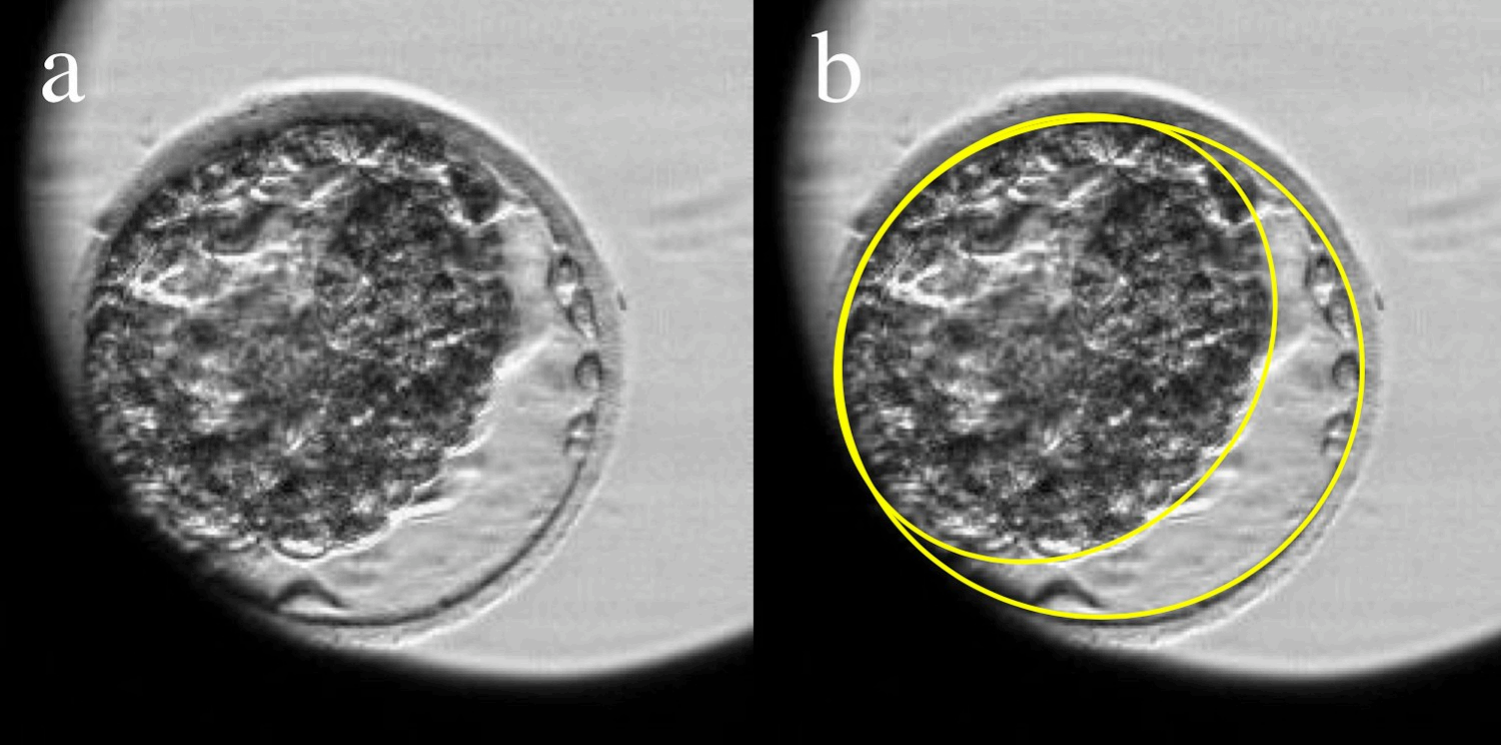
Definition of blastocyst contraction using TLC. **(a)** Original image of contracted blastocyst. **(b)** Surface of the contracted blastocyst analyzed using ‘Primo Vision analyzer’ drawing tools; the inner surface of the zona pellucida and outer surface of the TE are outlined in yellow. Contraction is defined as failure of these lines to coincide; in this image, the cells are 70% separated.

In previous reports, contraction was classified according to its extent [14]. However, we did not use this parameter to classify contraction because, when evaluating the separation rate of the zona pellucida surface and the contracted blastocyst surface, it was not possible to evaluate the actual extent of contraction using a single cross-section. Instead, contraction (rather than collapse) was defined as reported by Marcos *et al*. [10]: the cross-sectional area of the expanded blastocyst became smaller, and the blastocyst surface separated from the inner surface of the zona pellucida (Fig 2).

### Immunofluorescence

We performed chromatin, Oct4, and Cdx2 staining as described previously [15]. For Oct4 and Cdx2 staining, embryos were fixed in formaldehyde (3.7%) for 30 min at approximately 26 °C and then washed three times for 45 min in wash buffer-1 (Dulbecco’s phosphate-buffered saline [D-PBS] containing 0.1% bovine serum albumin [BSA]; Sigma—Aldrich, USA). Next, the embryos were permeabilized by placing them in wash buffer-2 (D-PBS containing 0.5% Triton X-100 [Sigma—Aldrich] and 0.1% BSA) for 30 min at approximately 26 °C. After washing three times in wash buffer-1, the embryos were blocked with D-PBS containing 3% BSA for 1 h at approximately 26 °C, and then incubated with anti-Oct3/4 primary antibodies (1: 50, sc-8628; Santa Cruz Biotechnology, USA) overnight at 4 °C. The next day, the embryos were washed three times in wash buffer-2, and then incubated with the secondary Alexa Fluor 647-conjugated donkey anti-goat antibodies (1: 100; Abcam, USA) for 30 min at approximately 26 °C. After washing three times in wash buffer-2, the embryos were incubated with anti-Cdx2 (ready-to-use, CDX2-88; Bio Genex, USA) for 1 h at approximately 26 °C, washed three times in wash buffer-2, and then incubated with secondary Alexa Fluor 488-conjugated goat anti-mouse IgG (1: 100; Invitrogen, USA) for 30 min at approximately 26 °C. Following another three washes, chromatin was stained with Hoechst 33342 (1: 1,000; Dojindo Molecular Technologies, Japan) for 20 min at approximately 26 °C.

### Imaging and cell counting

**Fig 3:**
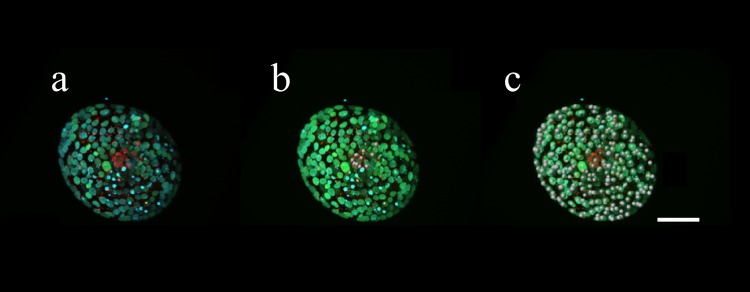
Number of ICM and TE cells counted using IMARIS imaging analysis software. **(a)** A 3D projection of an immunostained human expanded blastocyst (original image). **(b)** Number of ICM cells; red-stained nuclei were automatically marked with white spheres and counted. **(c)** Number of TE cells; green-stained nuclei were automatically marked and counted. Oct4 (red), Cdx2 (green), and chromatin (blue) nuclear staining. Scale bar, 70 μm.

Fully processed embryos were mounted in glass-bottom dishes (Matsunami Glass Ind. Ltd, Japan). Stained embryos were examined and measured using an LSM780 confocal laser-scanning microscope (Carl Zeiss AG, Germany). We used 20 × and 40 × oil-immersion objectives and 405, 488, and 633 nm lasers to obtain 1-μm-thick optical sections. The numbers of Oct4- and Cdx2- positive cells in the ICM and TE regions were counted as the number of ICM and TE cells, respectively. The number of ICM cells was set as 0 when the ICM was indistinguishable in the Non-Expanded group and the Cdx2-positive cells were counted as TE cells. Cell numbers were determined using IMARIS cell imaging software (Version 7.6; Bitplane AG, Switzerland) as described previously [16]. Chromatin, Oct4, or Cdx2-stained nuclei were determined either visually or by segmentation using 3D reconstructions with isosurface areas of 7–10 μm, depending on the nucleus size. Unmodified images were used for cell counting, and nuclei were considered positive if the intensity was above that of cytoplasmic staining (Fig 3). Co-staining was determined by marking spots in one channel and deleting that channel.

### Main outcome

We recorded the various morphokinetics obtained from each embryo, including the initial expansion rate, maximum expansion area, occurrence of hatching, and number of contractions, using TLC. We then investigated the relationship between these data and the number of ICM or TE cells using statistical methods. TE cells/maximal cross-sectional area was utilized as an index of density, as it was impossible to measure the surface area accurately when the blastocyst underwent contraction and hatching.

### Statistical analyses

All statistical analyses were performed using the Statistical Package for the Biosciences software (Version 9.67, Akita University, Japan). The Mann—Whitney U test was used to compare ages. Comparison of the proportion of each background parameter in the groups was examined using Fisher’s exact test or Chi-squared tests, depending on the sample size. Comparison of the number of ICM and TE cells for each parameter was performed using the Mann—Whitney U test. Spearman’s rank correlation test was used to examine correlations between the initial expansion rate and the numbers of ICM and TE cells, as well as the correlation between number of contractions in each embryo and number of TE cells per maximum expansion cross-sectional area. *P*-values < 0.05 were considered to be statistically significant differences.

## Results

### Characteristics of Expanded and Non-Expanded blastocysts

The Expanded group comprised 28 blastocysts, and the Non-Expanded group contained 10 blastocysts. Descriptive characteristics of the two blastocyst groups are shown in Table 1. There were significantly more 5 dpf blastocysts in the Expanded group. No significant differences were observed in other parameters between the two groups.

**Table 1:** Characteristics of Expanded and Non-Expanded blastocysts.

Parameters	Expanded group^a^ (n = 28)	Non-Expanded group^a^ (n = 10)	*p*
Age (mean ± SD)	35.2 ± 4.2	33.3 ± 4.6	0.2371
Diagnosis of infertility			0.7398
Male, n (%)	7 (25.0)	1 (10.0)	
Tubal, n (%)	2 (7.1)	1 (10.0)	
Endometriosis, n (%)	10 (35.7)	5 (50.0)	
Unexplained, n (%)	9 (32.1)	3 (30.0)	
Fertilization methods			0.8409
IVF, n (%)	24 (85.7)	9 (90.0)	
Intracytoplasmic sperm injection, n (%)	4 (14.3)	1 (10.0)	
Days post-fertilization (dpf)^b^			0.0178*
5 dpf, n (%)	11 (39.2)	0 (0)	
6 dpf, n (%)	17 (60.7)	10 (100)	
Embryo quality at vitrification^c^			0.7360
Good, n (%)	17 (60.7)	6 (60.0)	
Poor, n (%)	11 (39.2)	4 (40.0)	

^a^The Expanded group included blastocysts with a maximum expansion area of 5,000 μm^2^ or larger when observed using TLC for 22 h. The Non-Expanded group included blastocysts with maximum expansion area < 5,000 π μm^2^.

^b^Days post-fertilization (dpf) represents the number of days from fertilization to vitrification.

^c^Good embryos were defined as embryos with a Gardner grading of 4BB or higher. If an embryo failed to satisfy even one of the criteria for expansion, ICM, or TE, then it was defined as a poor embryo.

*p < 0.05 indicating significant difference

### Morphokinetics of the human embryos

In the Expanded group, the mean ± SD initial expansion rate was 14.9 ± 7.6 π μm^2^/min, and the maximum cross-sectional area was 10,137 ± 2,684 π μm^2^. Hatching occurred in 42.9% (12/28) of the blastocysts, and contraction occurred in 78.6% (22/28) of the blastocysts. The mean ± SD number of contractions was 2.0 ± 1.5. In the Non-Expanded group, hatching and contraction did not occur. Therefore, their morphokinetics are not discussed further. Three representative TLC videos are shown in S1–S3 Movies.

### Presence of biological differentiation markers

**Fig 4:**
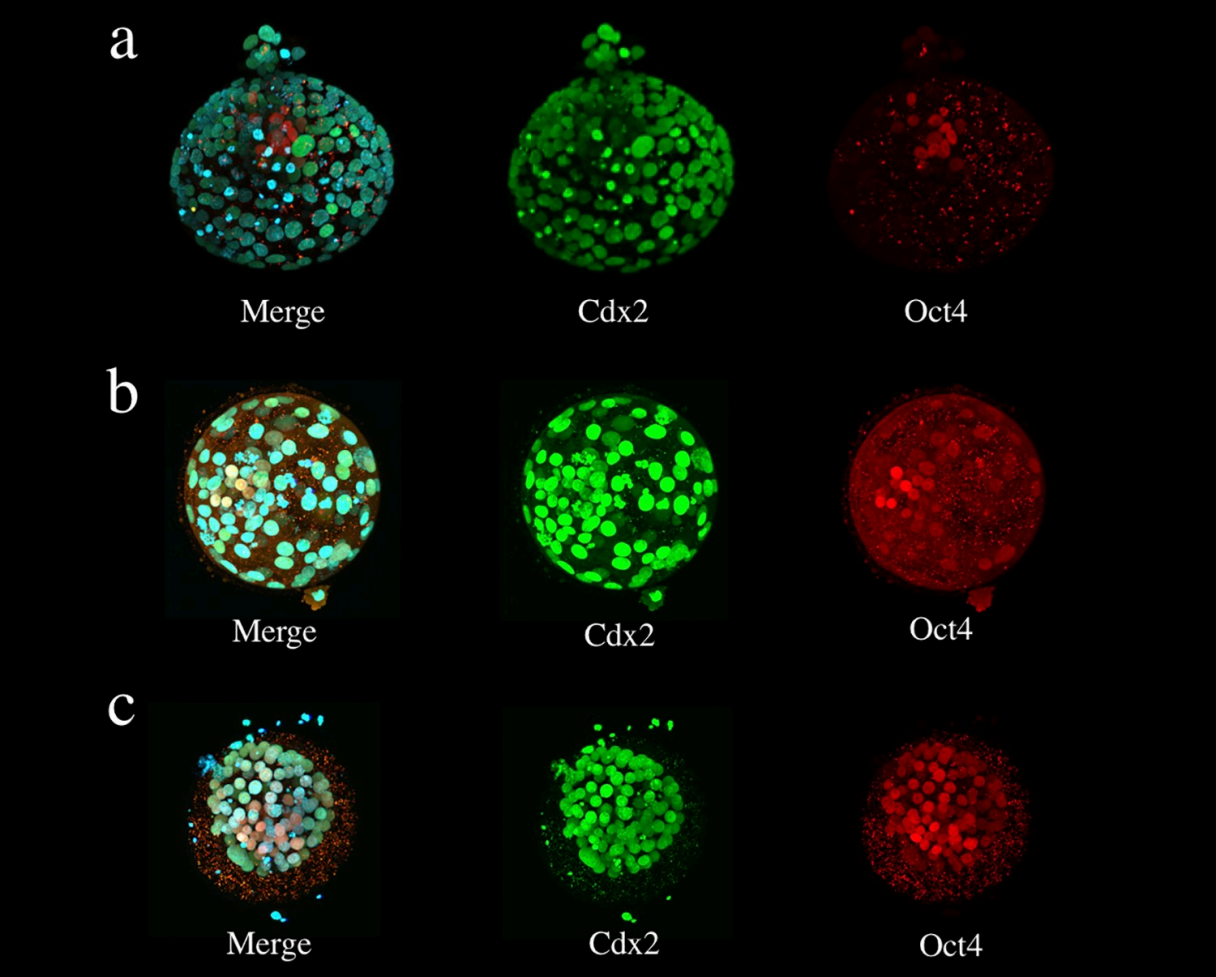
Presence patterns of Oct4 and Cdx2 in an immunostained human blastocyst. The immunostained samples were observed using confocal microscopy. **(a)** Blastocyst in the Expanded group; only Oct4-positive nuclei were found in the ICM and only Cdx2-positive nuclei were found in the TE. **(b)** Blastocyst in the Expanded group; double-positive (Oct4- and Cdx2-positive) nuclei were found in the ICM and TE regions. **(c)** Blastocyst in the Non-Expanded group; most of nuclei were double-positive (Oct4- and Cdx2-positive). Oct4 (red), Cdx2 (green), and chromatin (blue) nuclear staining.

All blastocysts in the Expanded group showed distinct ICMs and TEs. Immunostaining showed various patterns (Fig 4a and 4b). The Non-Expanded group exhibited frequent fragmentation (See S3 Movie), and the ICM was not found in 7 of the 10 blastocysts. The number of ICM cells was set as 0 for these embryos. The staining patterns of the blastocysts in the Non-Expanded group also varied, but most nuclei were double-positive (Oct4- and Cdx2-positive) (Fig 4c), although some differences were observed. These staining patterns were not related to the number of days from fertilization until vitrification.

### Number of ICM and TE cells

**Fig 5:**
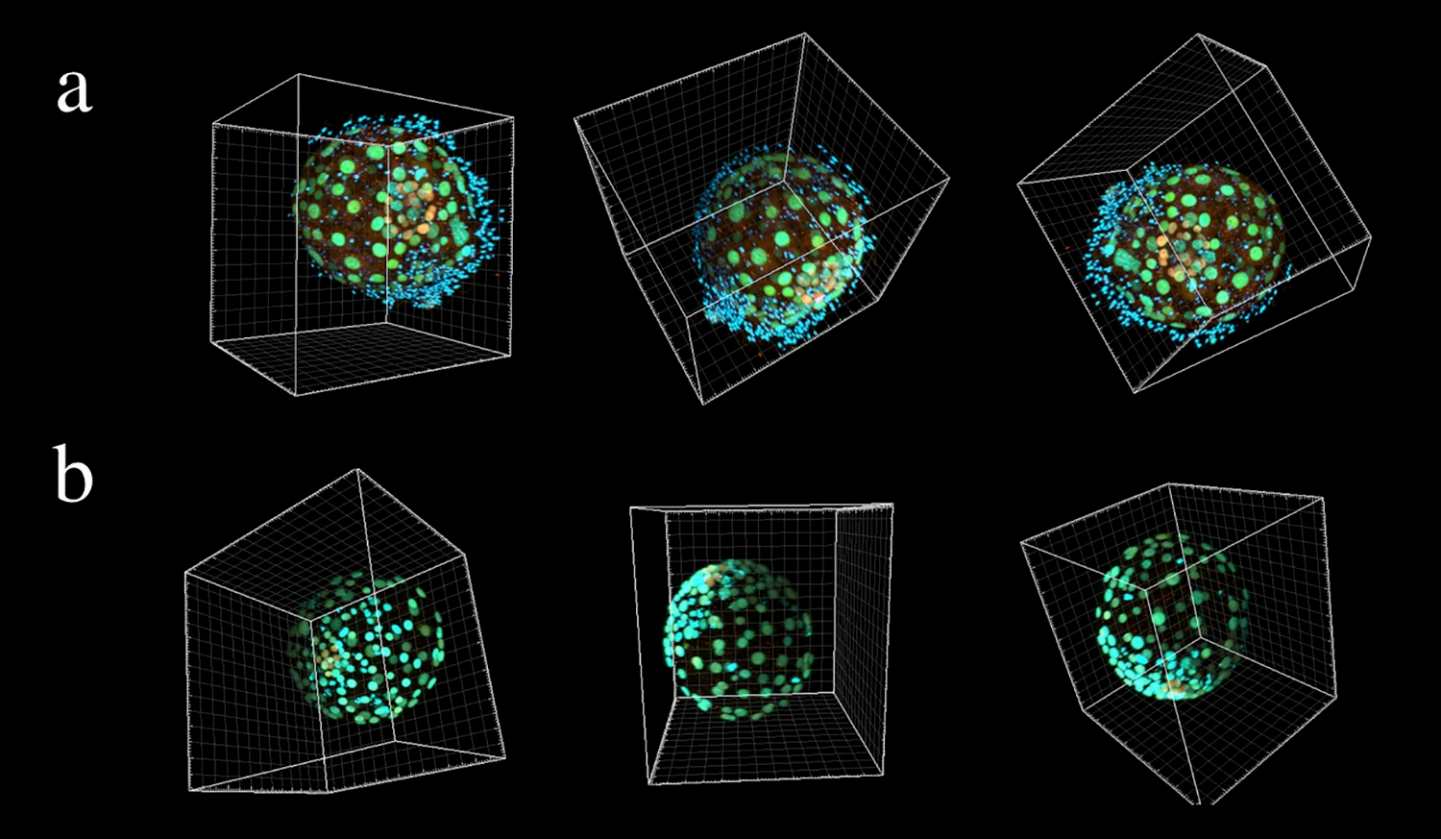
3D projection of an immunostained human expanded blastocyst observed from various directions. **(a)** Images of uniformly distributed TE cells. Many sperm heads are attached to zona pellucida. In the case of IVF, sperm heads may remain. **(b)** Images of unevenly distributed TE cells. Oct4 (red), Cdx2 (green), and chromatin (blue) nuclear staining.

**Fig 6:**
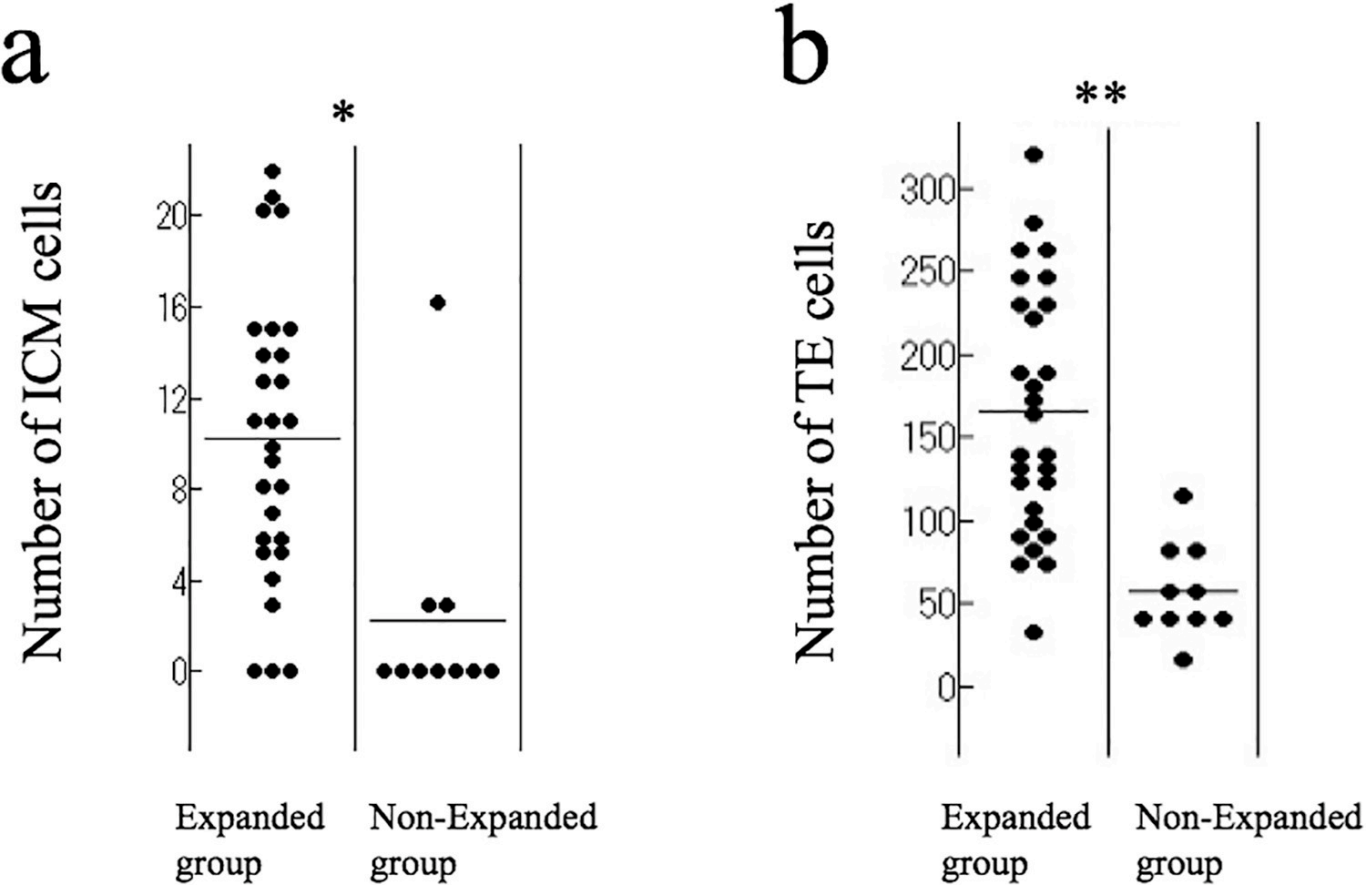
Number of ICM and TE cells in the Expanded (n = 28) and Non-Expanded groups (n = 10). **(a)** The number of ICM cells is significantly higher in the Expanded group than in the Non-Expanded group (*p = 0.0162, Mann-Whitney U test). Horizontal bar shows the mean. **(b)** The number of TE cells is significantly higher in the Expanded group than in the Non-Expanded group (**p < 0.0001, Mann-Whitney U test). Horizontal bar shows the mean.

Three-dimensional (3D) images of blastocysts revealed that some blastocysts had TE cells that were uniformly distributed, some blastocysts had unevenly distributed TE cells (Fig 5). Blastocysts in the Expanded group comprised a significantly higher number of ICM and TE cells than those in the Non-Expanded group (ICM: Expanded group 10.2 ± 6.3 [0–22] vs. Non-Expanded group 6.0 ± 12.3 [0–38], *p* = 0.0162; TE: Expanded group 165.7 ± 74.8 [35–321] vs. Non-Expanded group 57.0 ± 29.4 [16–119], *p* < 0.0001) (Fig 6). In the Expanded group, no significant correlation was observed between the number of ICM cells and age (age < 35 years, 10.1 ± 6.3 [0–20] vs. age ≥ 35 years, 10.3 ± 6.5 [0–22], *p* = 0.9633) or between the number of TE cells and age (age < 35 years, 178.9 ± 69.2 [77–278] vs. age ≥ 35 years, 152.5 ± 80.3 [35–321], *p* = 0.2802). At 5 dpf, blastocysts had a significantly higher number of ICM cells than the 6 dpf blastocysts (5 dpf 13.9 ± 6.1 [5–22] vs. 6 dpf 7.8 ± 5.3 [0–15], *p* = 0.0236). No significant relationship was found between the number of TE cells and the dpf (5 dpf 154.2 ± 54.4 [80–248] vs. 6 dpf 173.1 ± 86.2 [35–321], *p* = 0.6892).

### Correlation between the cell number and morphokinetics

**Fig 7:**
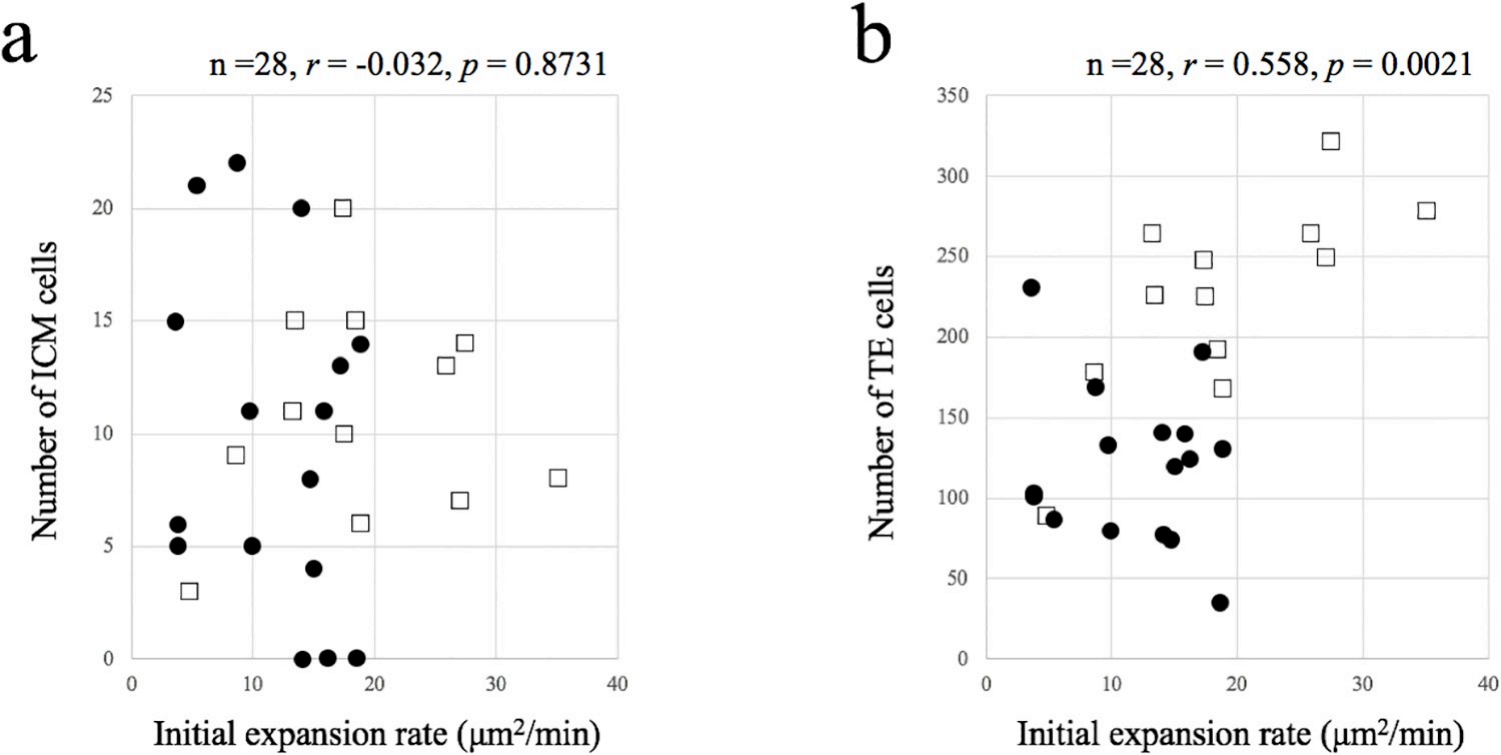
Correlation between the initial expansion rate and cell numbers. **(a)** Initial expansion rate and the number of ICM cells. **(b)** Initial expansion rate and the number of TE cells. Squares represent blastocysts that started hatching in culture within 22 h. Black circles represent those that did not start hatching.

**Fig 8:**
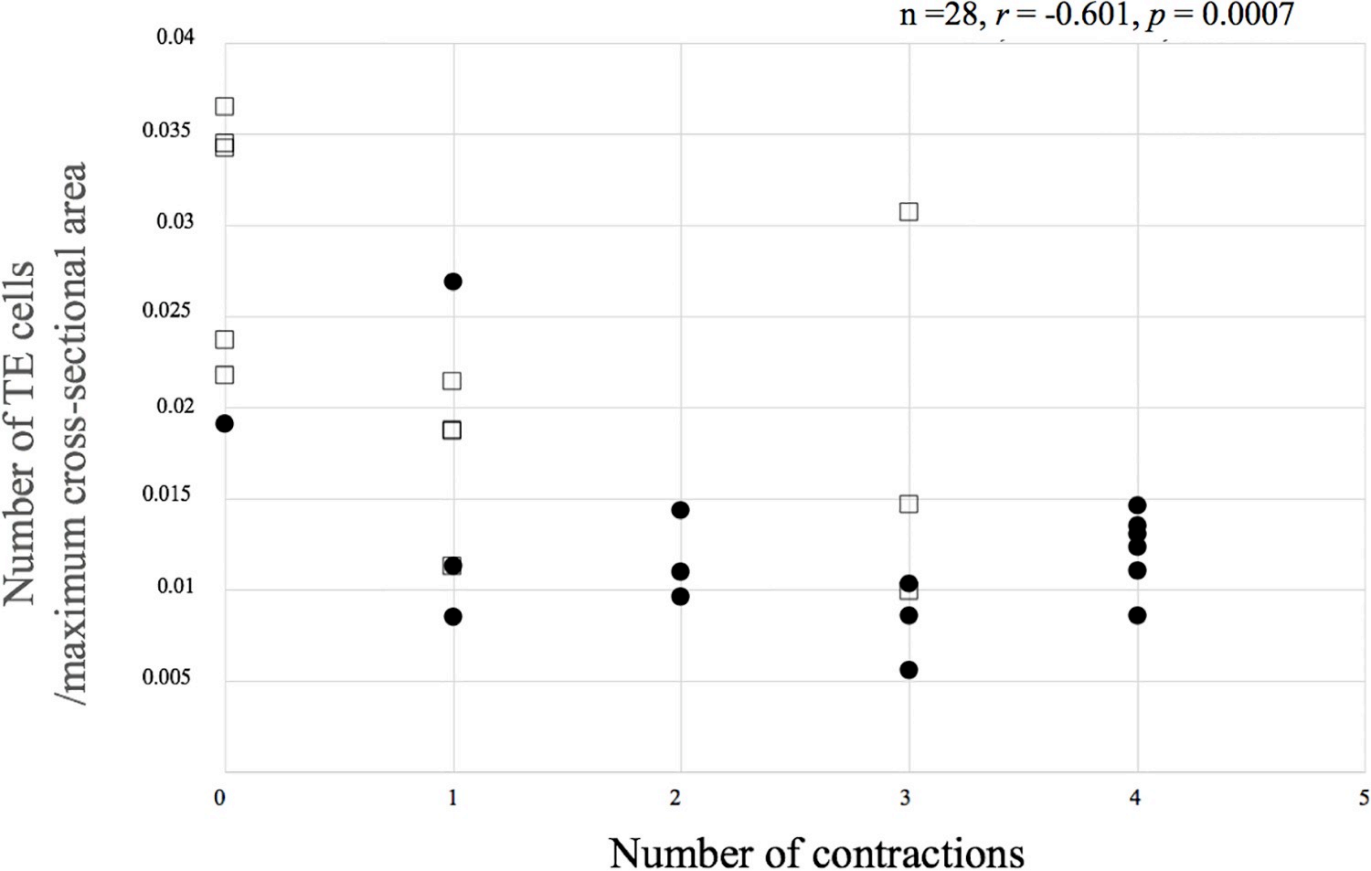
Correlation between the number of TE cells per unit of blastocyst maximum cross-sectional area and the number of contractions. Squares represent blastocysts that started hatching in culture within 22 h. Black circles represent those that did not start hatching.

**Fig 9:**
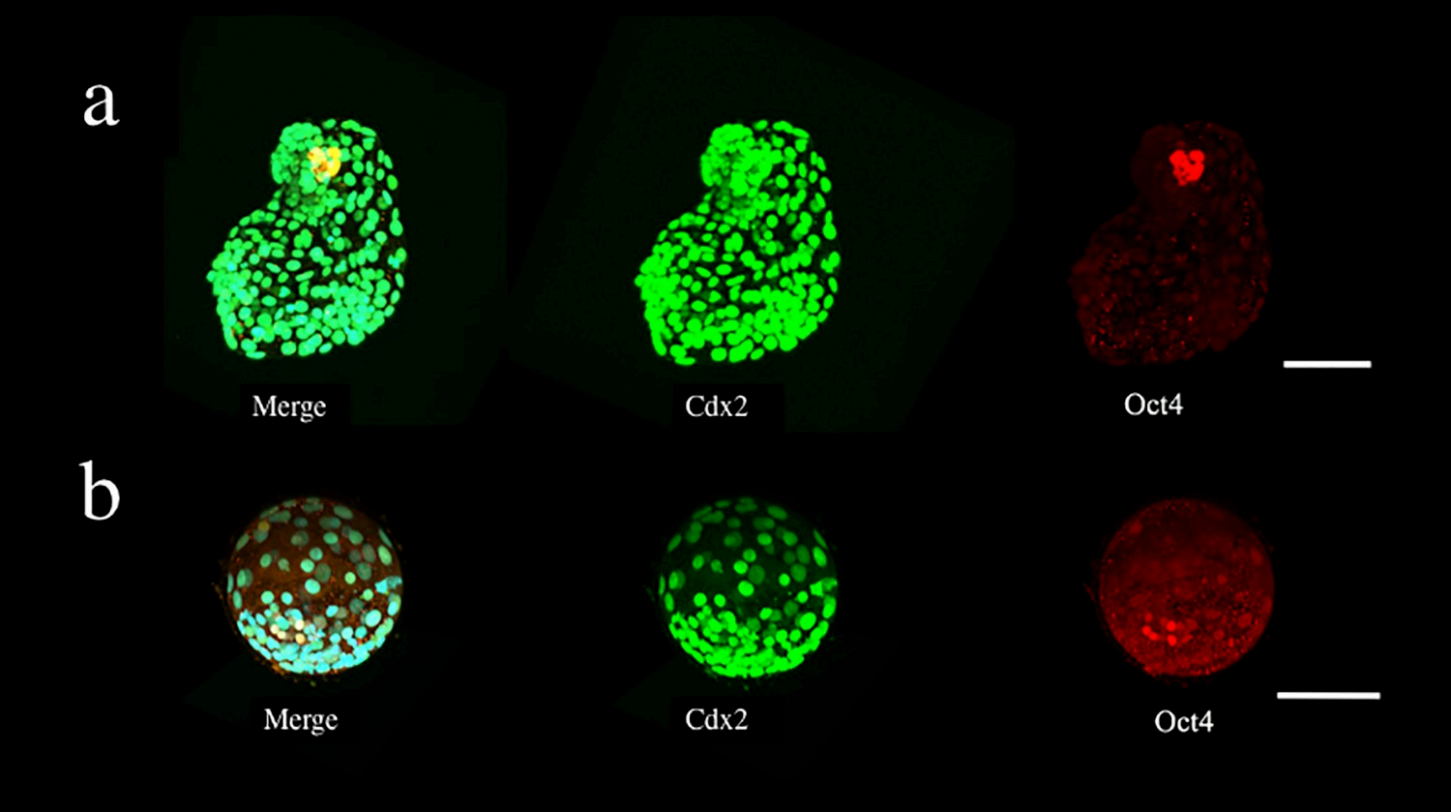
Representative images of human blastocysts with high (a) or low (b) numbers of TE cells per maximum expansion cross-sectional area observed using confocal microscopy. **(a)** Blastocyst fixed in hatching stage. **(b)** Blastocyst fixed in expanded stage. Oct4 (red), Cdx2 (green), and chromatin (blue) nuclear staining. Scale bar, 100 μm.

No correlation was detected between the number of ICM cells and the initial expansion rate (Fig 7a). There was a significant positive correlation between the number of TE cells and the initial expansion rate (*r* = 0.558, *p* = 0.0021) (Fig 7b). There was a significant negative correlation between the number of contractions for each embryo and number of TE cells per maximum expansion cross-sectional area (*r* = –0.601, *p* = 0.0007) (Fig 8). Fig 9 shows images of blastocysts with high and low numbers of TE cells per maximum expansion cross-sectional area. For these cases, the TLC videos are shown in S1 and S2 Movies. No relationship between the number of ICM cells and the occurrence of hatching could be established (not hatching 9.7 ± 7.4 [0–22] vs. hatching 10.9 ± 4.7 [3–20], *p* = 0.6195). The blastocysts in which hatching occurred had significantly more TE cells than those without hatching (not hatching 121.1 ± 48.6 [35–231] vs. hatching 225.2 ± 61.2 [89–321], *p* < 0.0001).

Tables 2 and 3 show the relationships between morphokinetics and numbers of ICM and TE cells, stratified by age (< 35, ≥ 35 years) and dpf (5, 6). There was no correlation between morphokinetics and the number of ICM cells in any stratification. In contrast, a correlation was observed between morphokinetics and the number of TE cells in every stratification. However, similar results were obtained when not stratified by age and dpf. Table 4 shows the correlation between number of contractions for each embryo and the number of TE cells per maximum expansion cross-sectional area, stratified by age (< 35, ≥ 35 years) and dpf (5, 6). The same result as when it was not stratified was obtained for age < 35 years and 6 dpf. However, although no significant difference was observed when stratified by age ≥ 35 years and 5 dpf, there was a tendency toward negative correlation.

**Table 2:** Correlation between the number of ICM and TE cells, initial expansion rate, and hatching with maternal age (< 35 or ≥ 35).

	Number of ICM cells		Number of TE cells	
Age	< 35 (n = 14)	≥ 35 (n = 14)	< 35 (n = 14)	≥ 35 (n = 14)
Initial expansion rate^a^	*r* = 0.012 *p* = 0.9665	*r* = −0.083 *p* = 0.7781	*r* = 0.515 *p* = 0.0596	*r* = 0.610 *p* = 0.0205
Hatching or not	*p* = 0.3993	*p* = 0.8463	*p* = 0.0045	*p* = 0.0282

Spearman’s rank correlation test was used to examine the correlation between the initial expansion rate and the number of ICM and TE cells. The numbers of ICM and TE cells were compared using the Mann—Whitney U test.

^a^The initial expansion rate indicates the rate up to 4 h after starting the imaging.

**Table 3:** Correlations between the dpf and the numbers of ICM and TE cells, initial expansion rate, and hatching.

	Number of ICM cells		Number of TE cells	
Days post-fertilization (dpf)^a^	5 dpf (n = 11)	6 dpf (n = 17)	5 dpf (n = 11)	6 dpf (n = 17)
Initial expansion rate^b^	*r* = 0.227 *p* = 0.5016	*r* = 0.189 *p* = 0.4686	*r* = 0.661 *p* = 0.0267	*r* = 0.535 *p* = 0.0271
Hatching or not	*p* = 0.9183	*p* = 0.1611	*p* = 0.0247	*p* = 0.0070

Spearman’s rank correlation test was used to examine the correlation between the initial expansion rate and the number of ICM and TE cells. The numbers of ICM cells and TE cells were compared using the Mann—Whitney U test.

^a^Days post-fertilization (dpf) is the number of days from fertilization to vitrification.

^b^The initial expansion rate indicates the rate up to 4 h after starting the imaging.

**Table 4:** Correlation between the number of contractions in each embryo and the number of TE cells per maximum expansion cross-sectional area with age (< 35 or ≥ 35) and dpf (5 or 6).

	< 35 (n = 14)	≥ 35 (n = 14)	5 dpf (n = 11)	6 dpf (n = 17)
Number of contractions Number of TE cells per maximal cross-sectional area	*r* = −0.819 *p* = 0.0003	*r* = −0.362 *p* = 0.2028	*r* = −0.097 *p* = 0.7775	*r* = −0.682 *p* = 0.0026

Spearman’s rank correlation test was used to examine the correlation between the number of contractions in each embryo and the number of TE cells per maximum expansion cross-sectional area.

Days post-fertilization (dpf) represents the number of days from fertilization to vitrification.

## Discussion

As Gardner reported, blastocysts in the Expanded group comprised a large number of ICM and TE cells [1]. In this study, we clarified the morphokinetics of frozen-thawed human blastocysts and demonstrated that the presence of re-expansion, initial expansion rate, occurrence of contraction, and hatching were related to the number of TE cells. These results were almost the same for each stratification with age (< 35 or ≥ 35) and dpf (5 or 6), with no observed influence of age and dpf.

A previous report showed partial counting of the number of TE cells in human blastocysts in cross-sections [3]. It became clear that TE cells were not always uniformly located; however, by constructing 3D images, it was possible to count the number of TE cells more accurately. The present study is one of the few to have counted all cells in human blastocysts. A report from 1989 showed that the mean numbers of TE cells ± SD in 5-, 6-, and 7-dpf human blastocysts were 37.9 ± 6.0, 40.3 ± 5.0, and 80.6 ± 15.2, respectively [16]. Here, we counted more TE cells than those in the previous report. This difference might be attributed to an improved culture environment or more precise counting methods. A study from 2013 used IMARIS (the same technology employed in this study) to count the number of TE cells in a human embryo [17], and the cell number was approximately the same as that found in this study. However, the earlier study did not focus on the relationship between cell number and blastocyst morphokinetics, but rather on the timing of gene expression in human blastocysts relative to that in mice. The results of the present study revealed that the blastocyst morphokinetics depended on the number of TE cells.

In the frozen-thawed embryos, fast re-expansion (within a few hours of thawing) is a significant indicator of ART outcome [5,18]. In addition, low blastomere loss is related to rapid re-expansion [5]. The results of this study demonstrated a positive correlation between the number of TE cells and expansion rate up to 4 h post-thawing. Based on the previous reports [19, 20], we hypothesize the following mechanism to explain the correlation between increased expansion rates and higher numbers of TE cells. The fertilized egg undergoes repeated cell divisions and becomes a blastocyst by differentiating into an ICM and TE via induction of Hippo signaling [12]. Initially, tight junctions and adherens junctions are formed between the TE cells [21]. The Na^+^/K^+^-ATPase present in the TE forms an ion gradient between the inside and the outside of the embryo. The aquaporin channel transports water into the blastocoel cavity following this concentration gradient, thus forming the blastocoel [22]. We suggest that in the presence of many TE cells, the amount of water drawn into the cavity increases, thereby increasing the water volume drawn into the blastocoel and accelerating the expansion rate. Post-thawing blastomere loss leads to a reduction in the normal number of TE cells, which may affect the re-expansion rate. Differentiation of blastocysts with sufficient TE cells into a future placenta would be beneficial for both implantation and the prognosis of a subsequent pregnancy.

There are reports that ICM morphological evaluation is effective for embryo selection [23]. In this study, the number of ICM cells was significantly larger in the Expanded group. The 6 dpf blastocysts had fewer ICM cells than the 5 dpf blastocysts. We initially hypothesized that this could be because at this stage, Oct4 expression disappears and the NANOG transcription factor is expressed at this stage [17]; however Oct4-negative and DAPI-positive nuclei were not found in the ICM. Therefore, the reason for this remains unknown. Further, the number of ICM cells was not related to the occurrence of hatching or other morphokinetic variables. This finding does not completely contradict the relationship between morphological evaluation of the ICM and future pregnancy or implantation rates. Further investigation is required to evaluate the ICM, which forms the future embryo.

The relationship between contraction and outcome of ART has been reported. In mouse embryos, contraction with a volume reduction below 20% is beneficial for hatching, whereas a contraction of 20% or more correlates with a low hatching rate [14]. In 2015, Marcos *et al*. reported that in humans, collapse correlated with a reduced pregnancy rate [10]. However, in 2016, Bodri *et al*. reported that collapse was not an indicator of ART outcome [11]; hence, this remains an unresolved topic. Contraction may change the embryo morphology by breaking down the adhesive state between cells, thereby inhibiting intercellular signaling and adversely affecting embryonic development. Currently, it is unknown whether contraction adversely affects the embryo or whether this tends to occur more readily in poor embryos.

This study focused on the TE cell number and showed that a lower number of TE cells per maximum expansion cross-sectional area correlated with more frequent contractions. Blastocysts with fewer TE cells per cross-sectional area exhibited greater distances between nuclei than those with more TE cells. We presume that when the blastocysts expand with fewer TE cells, the cellular junctions become physically prone to collapse because of an unknown mechanism. There is a possibility that distribution of TE cells may also be involved in this. In the current study, TE cells were variously distributed. Since TE cell distribution could be observed only at fixed timings, the distribution of TE cells at various timepoints, such as just before contraction, is not known; this needs to be addressed in future using techniques of live cell imaging [24].

The hatched blastocysts had more TE cells. Sathananthan H reported on hatching mechanism in 2003 [25]. It is important that the blastocyst is sufficiently expanded to stretch the zona pellucida thinly. When the number of TE cells is large, they can be expanded sufficiently without contraction, which would be advantageous for hatching.

The reduced number of TE cells may be attributable to the failure of cell division owing to organelle abnormalities [26,27], chromosomal abnormalities such as aneuploidy [28,29], and cell death. The latter has been reported to occur because of apoptosis [30] and damage caused by freezing and thawing [31]. Shimoda *et al*. have also reported on the damage caused by the freezing process [32]. In addition, advanced maternal age negatively affects embryo development [33]. In this study, we could not examine these individual causes. Furthermore, all 38 embryos had reached a Gardner expansion grade of 3 or higher at the time they were frozen; however, 10 of these embryos could not re-expand fully. This might be because of the effects of the freezing and thawing processes. Establishing a better freezing and thawing process and culturing environment may improve ART outcomes and embryo quality.

The expression patterns of Oct4 and Cdx2 are species-specific; however, Niakan and Eggan [17] reported that in human embryos, 3 dpf at the 8-cell stage, all blastomeres start to express Oct4. The blastocoel starts to form, and the expression of Cdx2 starts at 5 dpf. Initially, both Oct4 and Cdx2 are expressed in a single blastomere; however, each of these factors suppresses the other. At 6 dpf, when 128–256 cells are present, the expression of Oct4 is localized to the ICM. Gradually, only Cdx2 is expressed in the TE. The surrounding environment acts as a signal for the expression of these transcription factors [12]. In mice, cells that had once expressed Oct4 in the ICM start to express Cdx2 when moved to the TE region, suggesting that this expression is site-dependent [34]. Depending on the environment, TE cells in human expanded blastocysts can express the pluripotent marker NANOG, which is expressed by ICM cells [35]. In this study, blastocysts formed at 5–6 dpf; however, various Oct4 and Cdx2 distribution patterns were seen in each embryo. Notably, many of the fully re-expanded embryos had Cdx2-positive TE cells that also weakly stained for Oct4, making them double-positive. All cells in some non-expanded embryos were double-positive, which suggests that even at this stage, the re-expression of transcription factors depends on the surrounding environment.

This study had certain limitations. The sample number was small as only surplus human embryos were used because of ethical restrictions. Furthermore, we were unable to evaluate the cell number at the start of hatching and at the point of contraction, and we could not verify the prognosis, had the blastocysts been transferred.

Nevertheless, this is the first report to demonstrate the use of diversified human embryos, rather than the commonly used mouse or rabbit embryos, to show that different frozen-thawed blastocyst morphokinetic behavior reflects the number of TE cells. The rapid re-expansion up to 4 h post-thawing reflected the high number of TE cells. Conversely, a small maximum expansion cross-sectional area or repeated contractions reflected a small number of TE cells. Establishing a relationship between the number of TE cells and ART outcome will allow the morphokinetics observed using TLC to become a more effective indicator for selecting good embryos.

## Supporting information

S1 Movie Blastocyst expansion and hatching initiation, with no contraction. (MP4)

S2 Movie Blastocyst repeating expansion and contraction, with no hatching initiation. (MP4)

S3 Movie Blastocyst with no expansion and many fragmentations. (MP4)
